# Dispersive Full‐Channel Jones Matrix Modulation in Elliptical Polarization Bases via a Single‐Layered Metasurface

**DOI:** 10.1002/advs.202521354

**Published:** 2025-12-14

**Authors:** Hairong He, Guangtao Cao, Shuhao Zhang, Hui Xu, Zhiquan Chen, Meiyu Peng, Yuting Jiang, Yueqiang Hu, Hui Jing, Huigao Duan, Hui Yang

**Affiliations:** ^1^ Key Laboratory of Low‐Dimensional Quantum Structures and Quantum Control of Ministry of Education Department of Physics Hunan Normal University Changsha 410081 P. R. China; ^2^ Key Laboratory of Physics and Devices in Post‐Moore Era College of Hunan Province Changsha 410081 P. R. China; ^3^ School of Physics and Electronic Sciences Changsha University of Science and Technology Changsha 410004 P. R. China; ^4^ School of Microelectronics and Physics Hunan University of Technology and Business Changsha 410205 P. R. China; ^5^ National Research Center for High‐Efficiency Grinding College of Mechanical and Vehicle Engineering Hunan University Changsha 410082 P. R. China

**Keywords:** dispersive Jones matrix, metasurface, optical encryption, wavelength and polarization multiplexing

## Abstract

Polarization and wavelength multiplexing are two fundamental and extensively utilized techniques for enhancing capacity in multi‐functional meta‐optics. Despite the fact that existing works have pushed the channel numbers of Jones matrix (i.e., polarization) to their upper limit, the realization of wavelength‐multiplexed full‐channel Jones matrix manipulation remains a formidable challenge. Here, a general strategy is proposed to implement dispersive full‐channel Jones matrix modulation via a single‐layered metasurface. By synergistic dispersion engineering and polarization bases transformation, the proposed metasurface enables simultaneous modulation of all four Jones matrix channels across multiple wavelengths. This strategy, counterintuitively, unlocks the fundamental upper limit channel imposed by conventional linear or circular polarization bases. As a proof of concept, a dual‐wavelength and four‐elliptical‐polarization multiplexed meta‐hologram is experimentally and numerically demonstrated, which is capable of achieving eight color vectorial holographic images. By extending it to 3D space with sixteen vectorial holographic images, a dual‐key‐space convolutional encryption platform is further demonstrated, which is able to achieve parallel optical information transmission with ultra‐high security. The proposed paradigm overcomes the intrinsic channel‐capacity limitations of single‐layer metasurfaces, opening new pathways for the development of advanced multi‐functional meta‐devices and high‐security information processing systems.

## Graphical abstract

This work presents a dispersive full‐channel Jones matrix modulation strategy using single‐layer metasurface. By synergizing wavelength dispersion engineering with elliptical polarization bases, independent control of four Jones matrix channels is achieved across multiple wavelengths. As proof of concept, a dual‐wavelength meta‐hologram reconstructs eight color vectorial images, and a dual‐key‐space convolutional encryption platform is demonstrated for high‐security parallel optical information transmission.

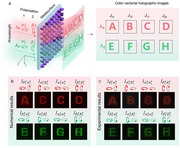

## 1 Introduction

Polarization, amplitude, phase, and wavelength are fundamental properties of electromagnetic waves, and controlling them completely and arbitrarily lays the basis for multi‐functional optical devices. Metasurfaces, artificial subwavelength scale nanostructures in a 2D plane, enable manipulation of the abundant degrees of freedom (DoFs) of light such as phase, amplitude, and polarization with unprecedented capacities.^[^ ^1^, ^2^ ^]^ By skillfully tailoring the topology, size, and spatial arrangement of subwavelength nanostructures, the optical responses of the metasurfaces can be manipulated in an almost arbitrary way.^[^ ^3^, ^4^, ^5^ ^]^ As a result, the wavefront of incident light can be shaped at will, promising to unlock exceptional functionalities beyond the conventional bulky counterparts. In recent years, metasurfaces have been extensively explored for diverse meta‐devices, such as meta‐holograms,^[^ ^6^, ^7^, ^8^ ^]^ metalenses,^[^ ^9^, ^10^, ^11^ ^]^ vector beam generators,^[^ ^12^, ^13^, ^14^ ^]^ and polarimeters,^[^ ^15^, ^16^, ^17^, ^18^ ^]^ allowing versatile applications in various fields.

Polarization, which characterizes the vectorial nature of the oscillating electric field, has been extensively explored in metasurfaces for applications ranging from imaging^[^ ^19^, ^20^ ^]^ and encryption^[^ ^21^, ^22^ ^]^ to quantum operations.^[^ ^23^ ^]^ In general, the manipulation of polarization by metasurfaces can be described by a 2 × 2 matrix, i.e., the Jones matrix, which has four elements that are respectively composed of the amplitude and phase terms.^[^ ^24^ ^]^ Mathematically, the Jones matrix has four independent channels along with eight DoFs. For a single‐layer metasurface, the off‐diagonal elements of the Jones matrix are identical due to the structure's in‐plane mirror symmetry, and the number of the channels is reduced to three.^[^ ^25^, ^26^ ^]^ Actually, almost all the metasurfaces‐enabled functionalities can be categorized into the manipulation of different channels in the Jones matrix.^[^ ^27^, ^28^, ^29^, ^30^, ^31^, ^32^ ^]^ To date, numerous efforts have been made to extend the number of manageable channels, and an upper limit of three for the single‐layer metasurface has been achieved.^[^ ^33^ ^]^ By cascading two‐layer metasurfaces, the ultimate goal of manipulating the four Jones matrix channels has been demonstrated.^[^ ^24^, ^32^, ^34^ ^]^ Very recently, by employing the elliptical polarization bases, all four Jones matrix channels have been effectively decoupled via a single‐layered metasurface.^[^ ^35^ ^]^ Meanwhile, the full‐parametric modulation of the Jones matrix has also been achieved on an integrated photonic platform using an on‐chip metasurface design, showcasing its potential for high‐capacity multiplexing in guided‐wave radiation.^[^ ^36^ ^]^ Despite these achievements, the realization of wavelength‐multiplexed full‐channel Jones matrix manipulation​ in a free‐space​ configuration via a single‐layered metasurface​ remains a formidable challenge, which makes notable constraints in practical applications such as high‐capacity optical display and data storage. To break this inherent constraint, exploiting the residual dimensions of photon on this basis, would be an excellent choice.

Wavelength, a fundamental photon dimension that correlates to the energy level transitions, has been widely utilized for wavelength‐division multiplexing in optical fiber communications. Correspondingly, great efforts have been made to explore the wavelength dimension in metasurface design, including multi‐wavelength metalens,^[^ ^9^, ^10^, ^37^ ^]^ color nano‐printing and holograms,^[^ ^38^, ^39^, ^40^, ^41^ ^]^ color vortex beam^[^ ^42^ ^]^ and so forth. Therefore, the wavelength dimension can be added to the Jones matrix to break the inherent upper limit on multiplexing channels. In linear optics, all the previously demonstrated wavelength‐dependent functionalities can be regarded as the synthesis of the wavelength dimension and the Jones matrix with a specific number of channels. Initial efforts have focused on single‐polarization channel full‐color nano‐printing or hologram,^[^ ^43^, ^44^, ^45^ ^]^ which can be attributed to dispersive Jones matrix with one channel. Subsequently, the dispersive Jones matrix with two channels has been obtained. Typical instances are the polarization‐control dual‐channel achromatic metalenses and meta‐holograms enabled by the dispersive phase control of the two Jones matrix elements.^[^ ^20^, ^46^ ^]^ Very recently, the dispersive phase control of three Jones matrix channels has been demonstrated via the gradient‐based optimization algorithm using deep neural network.^[^ ^47^, ^48^ ^]^ Besides, by utilizing polarization states as information carriers, an on‐demand, reconfigurable mapping between incident polarization vectors and output signals on the dispersive Poincaré sphere have been established for optical vector analog computing.^[^ ^49^ ^]^ Although the great achievements in expanding the number of multiplexing channel, the dispersive Jones matrix with full‐channel modulation via a single‐layered metasurface, has not yet been realized.

Here, counterintuitively, we achieve dispersive full‐channel Jones matrix modulation via a single‐layer metasurface. This general strategy overcomes the intrinsic three‐channel limit of planar metasurfaces with linear or circular polarization bases. By synergistic wavelength dispersion engineering and polarization base transformation, the proposed metasurface enables simultaneous and independent modulation of all four Jones matrix channels across multiple wavelengths. For this, polyatomic TiO_2_ elliptical nanoblocks are elaborately designed to achieve elliptical polarization decoupling and wavelength dispersion engineering. To elucidate our concept, we conduct comprehensive theoretical calculations to establish the intricate relationship between the four channels of Jones matrix and their corresponding orthogonal polarization bases. As a proof of concept, we numerically and experimentally demonstrate a meta‐hologram with eight color vectorial holographic images via dual‐wavelength and four‐elliptical‐polarization multiplexing. To further verify the proposed strategy, we extend the color vectorial meta‐hologram to 3D space, with sixteen holographic images exhibited at two imaging planes. Using the 3D color vectorial meta‐hologram, we construct a dual‐key‐space convolutional encryption platform that merges physical optical keys with mathematical operations, enabling parallel optical information transmission with high security.

## 2 Principles and Metasurface Design

**Figure 1:**
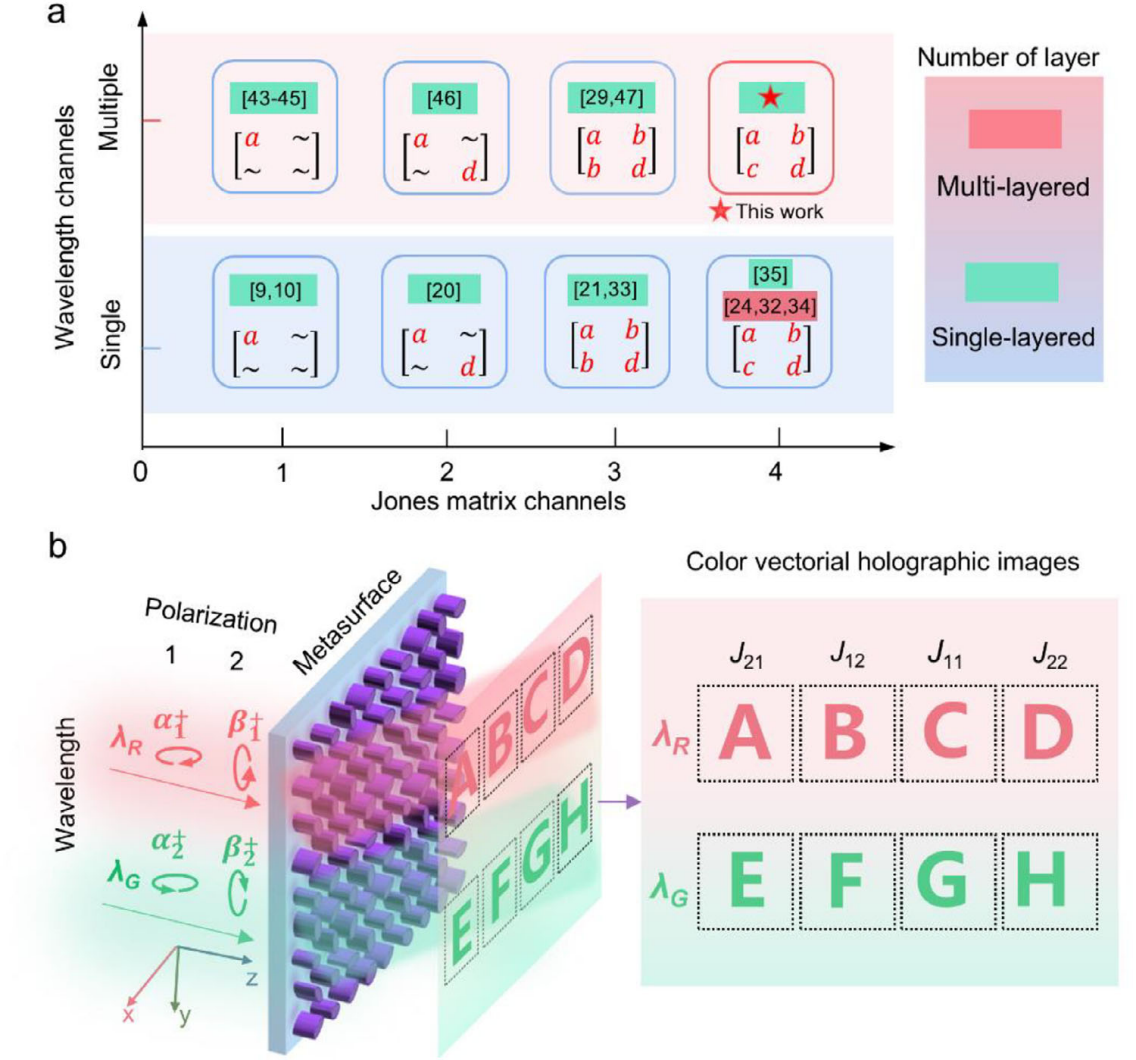
Schematic illustration of the dispersive full‐channel Jones matrix modulation via a single‐layered metasurface. a) A summary of wavelength and Jones matrix channels multiplexing using metasurfaces in the previous literatures. The multiplexing wavelengths and the number of metasurface layers are both separated as category of single and multiple. The red pentacle indicates our work, which realizes wavelength‐multiplexed full Jones matrix channels with a single‐layer metasurface. b) Schematic illustration of a representative metasurface capable of multiplexing four Jones matrix channels at two distinct wavelengths for holographic display. The metasurface enables eight channels, with all generated vectorial holographic images being independent with each other.

The schematic diagram of the dispersive full‐channel Jones matrix modulation based on a single‐layer metasurface is shown in **Figure** **1**. The metasurface is elaborately designed to simultaneously modulate and decouple the correlation of wavelength and Jones matrix channels. As a result, it enables simultaneous and independent modulation of all four Jones matrix channels across multiple wavelengths. Here, we demonstrate a metasurface with eight multiplexing channels (i.e., two wavelengths multiplexed with four elliptical polarization (EP) channels). As shown in Figure 1a, we summarize the wavelength and Jones matrix channels multiplexing meta‐devices reported in the previous literatures. The multiplexing wavelengths and number of metasurface layers are both separated as category of single or multiple. The red pentacle indicates our work, which is capable of achieving wavelength‐multiplexed full Jones matrix channels with a single‐layer metasurface. Figure 1b shows the wavelength and Jones matrix channels multiplexing meta‐hologram. Here, the EP states are used to unlock the upper limit of Jones matrix channels of the single‐layer metasurface. The metasurface supports four Jones matrix components (*J*_11_, *J*_12_, *J*_21_, and *J*_22_) and enables multiplexing across two wavelengths, specifically, red (R) and green (G) wavelengths (*λ*_R_ and *λ*_G_), with all generated vectorial holographic images being independent with each other.

**Figure 2:**
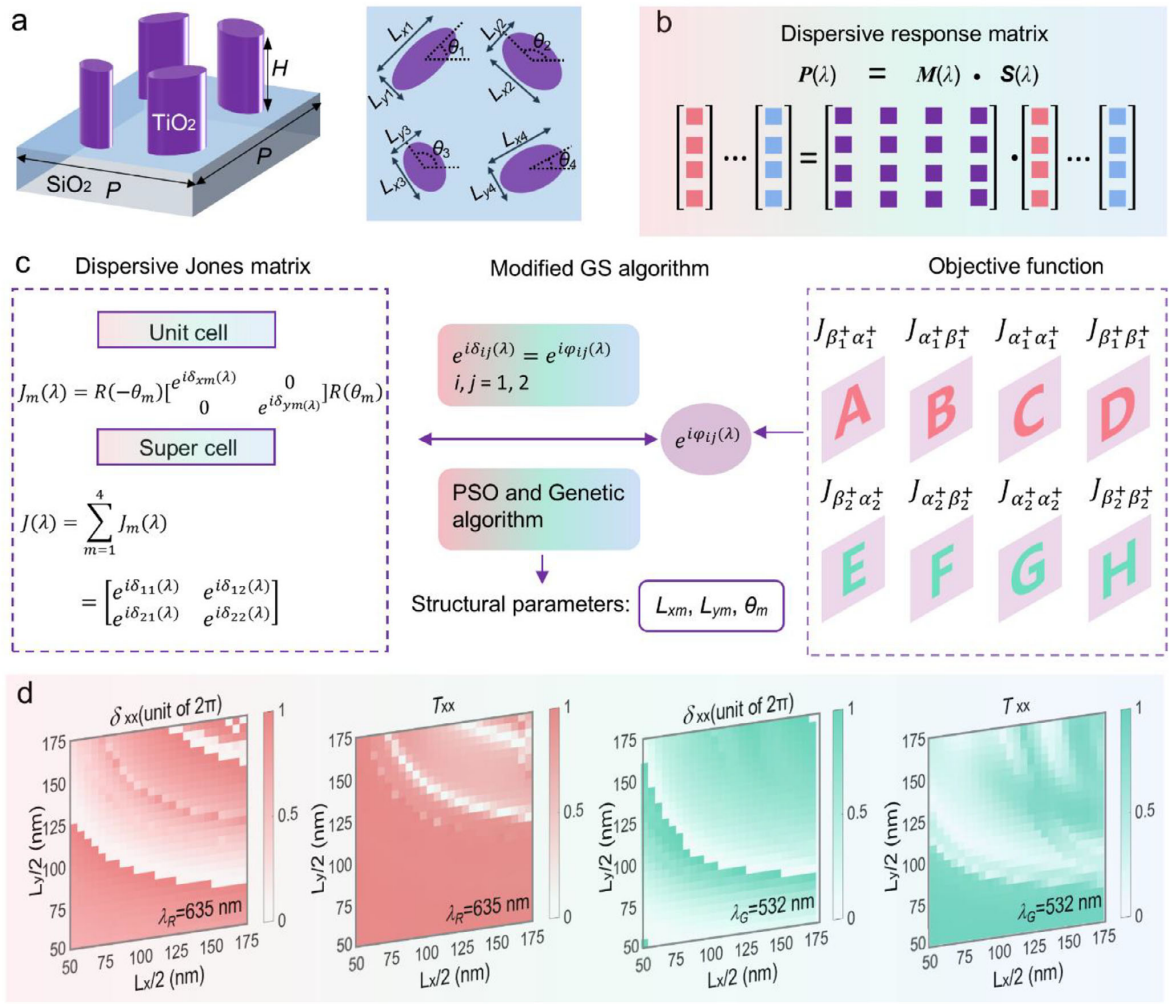
Schematic and design principle of the single‐layered dispersive Jones matrix full‐channel metasurface. a) Schematic of the super cell, which consists of four anisotropic meta‐atoms, from which one can see the detailed parameters. b) The optical response matrix of the designed dispersive metasurface. c) Flowchart of the design principle of dispersive full‐channel Jones matrix modulation with anisotropic metasurface. The calculated dispersive phase profiles are assigned to the anisotropic meta‐atoms via a custom‐developed integrated optimization approach that incorporates both the PSO algorithm and the GA. The wavelength‐multiplexed is typically demonstrated at two discrete wavelengths of *λ_R_* and *λ_G_*. d) The calculated phase shift and transmittance of the meta‐atom at two distinct wavelengths of *λ_R_* and *λ_G_*, along with the relationship between its long and short axes.

As illustrated in **Figure** **2**a, the polyatomic composite super cell is utilized to realize dispersive full‐channel Jones matrix modulation. The designed super cell consists of four TiO_2_ elliptical nanoblocks placed on a silica substrate. The side and top views of the super cell are presented in the left and right panels of Figure 2a, respectively. The detailed structural parameters include a lattice constant *P*, height *H*, and in‐plane dimensions of each meta‐atom (major axis *L_xm_*, minor axis *L_ym_* and the orientation angle *θ_m_*, where *m* represents the index of the four meta‐atoms). To reveal the physical mechanism, we start with the dispersive Jones matrix of an elliptic cylinder meta‐atom, which can be expressed as

(1) $$J\;\left(\lambda \right)=R\left(-\theta \right)\left[\begin{array}{cc} e^{i\delta_{x}\left(\lambda \right)} & \;0\\ 0 & \;e^{i\delta_{y}\left(\lambda \right)} \end{array}\right]R\left(\theta \right)$$

where *λ* is the working wavelength and *R*(*θ*) is the rotation matrix. *δ_x_* (*λ*) and *δ_y_* (*λ*) represent the propagation phase shifts under two orthogonal linearly polarized (LP) lights at wavelength *λ* along the meta‐atom's two symmetry axes. For the super cell that composed of four meta‐atoms, the final superimposed dispersive Jones matrix can be expressed as

(2) $$\begin{array}{c} J\;\left(\lambda \right)=\sum_{m=1}^{4}R\left(-\theta_{m}\right)\left[\begin{array}{cc} e^{i\delta_{xm}\left(\lambda \right)} & \;0\\ 0 & \;e^{i\delta_{ym}\left(\lambda \right)} \end{array}\right]R\left(\theta_{m}\right)=\left[\begin{array}{cc} e^{i\delta_{11}\left(\lambda \right)} & \;e^{i\delta_{12}\left(\lambda \right)}\\ e^{i\delta_{21}\left(\lambda \right)} & \;e^{i\delta_{22}\left(\lambda \right)} \end{array}\right] \end{array}$$

where *δ_ij_* (*λ*) (*i*, *j* = 1, 2) represents the phase shifts in the four channels of the final superimposed dispersive Jones matrix. Next, we consider the optical response of the super cell under an elliptical polarized incident light. For an arbitrary pair of orthogonal elliptical polarization states, |*α* ^+^ > and |*β* ^+^ >, which can be expressed in the orthogonal circular polarization bases as the form

(3) $$|\alpha^{+}\rangle =\left[\begin{array}{c} e^{i\psi }\cos\chi \\ e^{-i\psi }\sin\chi \end{array}\right];|\beta^{+}\rangle =\left[\begin{array}{c} -e^{i\psi }\sin\chi \\ e^{-i\psi }\cos\chi \end{array}\right]$$

where *ψ* and χ represent the azimuth angle and ellipticity of the polarization ellipse, respectively. The optical response for the full Jones matrix channels (i.e., the co/cross‐polarization channels) can be written as (see details in Note S1, Supporting Information)

(4) $$\begin{array}{ccc} \left[\begin{array}{c} P_{11}\left(\lambda \right)\\ P_{12}\left(\lambda \right)\\ \begin{array}{c} P_{21}\left(\lambda \right)\\ P_{22}\left(\lambda \right) \end{array} \end{array}\right] & = & \left[\begin{array}{cc} \begin{array}{cc} cos^{2}\left(\chi \right) & \;e^{-i2\psi }\frac{sin\left(2\chi \right)}{2}\\ -\frac{sin\left(2\chi \right)}{2} & \;cos^{2}\left(\chi \right) \end{array} & \;\begin{array}{cc} e^{i2\psi }\frac{sin\left(2\chi \right)}{2} & \;sin^{2}\left(\chi \right)\\ -e^{i2\psi }sin^{2}\left(\chi \right) & \;e^{i2\psi }\frac{sin\left(2\chi \right)}{2} \end{array}\\ \begin{array}{cc} -\frac{sin\left(2\chi \right)}{2} & \;-e^{-i2\psi }sin^{2}\left(\chi \right)\\ sin^{2}\left(\chi \right) & \;-e^{-i2\psi }\frac{sin\left(2\chi \right)}{2} \end{array} & \;\begin{array}{cc} e^{i2\psi }cos^{2}\left(\chi \right) & \;-\frac{sin\left(2\chi \right)}{2}\\ -e^{i2\psi }\frac{sin\left(2\chi \right)}{2} & \;cos^{2}\left(\chi \right) \end{array} \end{array}\right]\\ & & \times \;\left[\begin{array}{c} S_{11}\left(\lambda \right)\\ S_{12}\left(\lambda \right)\\ \begin{array}{c} S_{21}\left(\lambda \right)\\ S_{22}\left(\lambda \right) \end{array} \end{array}\right] \end{array}$$

This equation can be simplified as *P* (λ) =  *M*(λ) · *S*(λ), where *P* (λ) = [*P*_11_(λ),*P*_12_(λ),*P*_21_(λ),*P*_22_(λ)]^*T*^ , *S* (λ) = [*S*_11_(λ),*S*_12_(λ),*S*_21_(λ),*S*_22_(λ)]^*T*^ , and *M*(λ) is a 4 × 4 matrix. To visualized this, the optical response matrix for a meta‐device operates simultaneously at N discrete wavelengths can be expressed as shown in Figure 2.

As well known, introducing the wavelength DoF into the full Jones matrix channel would expand the available multiplexing channels. However, under such circumstances, Equation 4 does not yield an exact solution for the required polarization and wavelength multiplexed optical responses. Hence, without appropriate processing, there is inevitably crosstalk between the multiplexing channels. Nevertheless, with appropriate noising management, including introducing correlated noise via least‐squares solutions and uncorrelated random noise to break channel correlations,^[^ ^50^ ^]^ pre‐shaping the wavefront to locally approximate Fresnel diffraction as a Fourier transform at each image plane, and incorporating random phase distributions to exploit the orthogonality of high‐dimensional random vectors,^[^ ^51^ ^]^ crosstalk between inter channels and across depth planes can be effectively suppressed, enabling simultaneous polarization‐ and wavelength‐multiplexed modulation. Figure 2c presents the flowchart illustrating the design principle of dispersive full‐channel Jones matrix modulation based on a single‐layer anisotropic metasurface. The dispersive phase profiles of color full‐channel vectorial meta‐hologram are calculated by a modified Gerchberg–Saxton (GS) algorithm. The calculated dispersive phase profiles are used to construct the target dispersive Jones matrix. This target matrix is then mapped onto the anisotropic meta‐atoms via a custom‐developed integrated optimization approach, which combines both the Particle Swarm Optimization (PSO) algorithm and the Genetic Algorithm (GA). For the custom‐developed integrated optimization approach, PSO provides fast convergence, and GA ensures comprehensive global optimization, thereby collectively improving solution quality and avoiding local minima. The optimization process aims to minimize a predefined cost function ($F=\sum_{i=1}^{2}\sum_{j=1}^{2}\left|\;e^{\delta_{ij}\left(\lambda \right)}-e^{\varphi_{ij}\left(\lambda \right)}\right|$, where *φ* denotes the target phase) that quantifies the difference between the target Jones matrix and the achievable Jones matrix of the super cell of metasurface. This ensures that the designed meta‐atoms collectively satisfy the desired dispersive full‐channel modulation characteristics across the specified wavelengths. Subsequently, the meta‐atoms are elaborately designed to fulfill the desired modulation characteristics of the target Jones matrix. To investigate the transmission properties of the meta‐atoms, full‐wave electromagnetic simulations are performed using the finite‐difference time‐domain (FDTD) method. Figure 2d presents the simulated transmission phase shifts and amplitudes of the meta‐atoms under *x*‐linearly polarized incident light, showing variations with respect to the in‐plane dimensions (*L_x_* and *L_y_*) at two working wavelengths of *λ_R_* = 635 nm and *λ_G_* = 532 nm, respectively. The corresponding phase shifts and amplitudes under *y*‐linearly polarized incident light can be easily obtained by matrix transpose operation (see details in Note S2, Supporting Information). Consequently, the detailed geometric parameters of each meta‐atom, including in‐plane dimensions (*L_x_* and *L_y_*) and orientation angle (𝜃), at any coordinate (*x, y*) on the metasurface plane can be determined.

## 3 Dispersive Full‐Channel Jones Matrix Modulated Vectorial Holography

**Figure 3:**
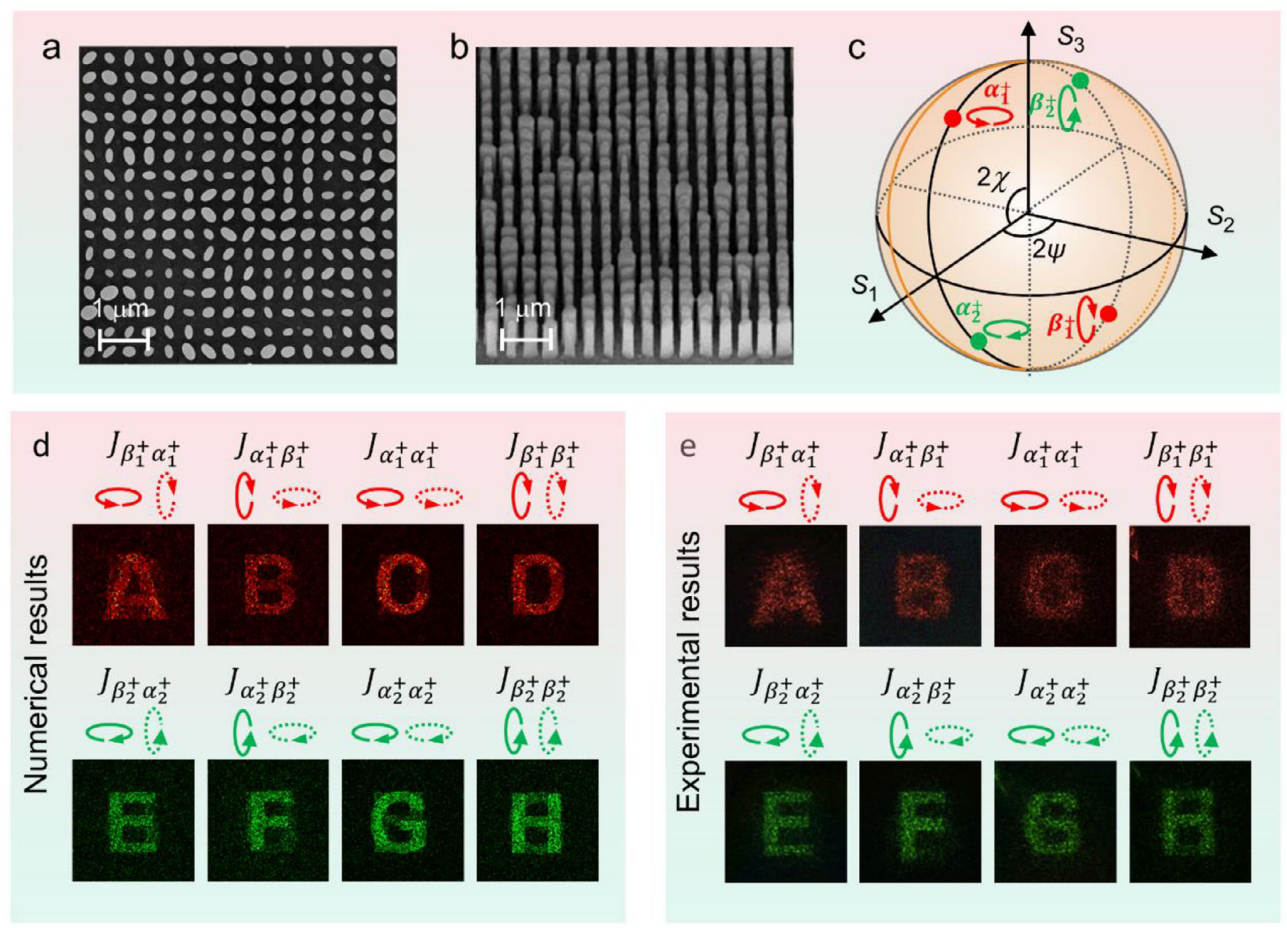
Implementation of dispersive full‐channel Jones matrix modulated vectorial holography. a) The top‐view SEM images of the fabricated metasurface. b) The oblique‐view SEM images of the fabricated metasurface. c) The investigated EP states represented as certain points on the surface of the Poincaré sphere. d,e) The numerical and experimental reconstruction results for the eight independent vectorial holographic channels of the designed meta‐device. The columns correspond to the four Jones matrix channels, while the rows correspond to the two operating wavelengths, *λ_R_* and *λ_G_*. Each holographic image is reconstructed under a specific combination of incident wavelength, input polarization state (indicated at upper left with solid line), and analyzed output polarization state (indicated at upper right with dotted line), as defined by its corresponding Jones matrix channel.

To validate the proposed dispersive full‐channel Jones matrix modulation strategy, we choose a multi‐channel color vectorial holography as a typical example. The metasurface was engineered to encode eight distinct target holographic images, labeled from “A” to “H”. The corresponding manipulation DoFs are the combination of two distinct wavelengths (*λ_R_ =* 635 nm and *λ_G_ =* 532 nm) and four pairs of orthogonal elliptical polarization states that construct the four Jones matrix channels (*J*_11_, *J*_12_, *J*_21_, and *J*_22_). Achieving eight independent channels with high fidelity and minimal crosstalk represents a significant advancement. By initiating research with two wavelengths, the design, processing, and measurement procedures of dispersive full‐channel Jones Matrix modulation system can be systematically verified. This ensures the reliability and accuracy of each procedure before expanding to more wavelengths. For experimental validation, the metasurface sample was fabricated using established nanolithography techniques, including electron‐beam lithography and atomic layer deposition, and adhered to dry etching process (see fabrication details in tExperimental Section). **Figure** **3**a,b presents the top‐view and oblique‐view scanning electron microscopy (SEM) images of the fabricated metasurface, respectively. For experimental characterizing the optical performance, a customized optical setup was employed (see details in Note S3, Supporting Information).

Figure 3d,e presents the numerical and experimental results for all eight independent vectorial holographic channels of the designed meta‐device. At the working wavelength *λ_R_* = 635 nm, the incident polarization state is EP states $|\alpha_{1}^{+}$> = (*χ* = π/12, *ψ* = 0) or its orthogonal counterpart $|\beta_{1}^{+}$> = (*χ* = 5π/12, *ψ* = π/2). At the working wavelength *λ_G_* = 532 nm, the incident polarization state is EP states $|\alpha_{2}^{+}$> = (*χ* = 5π/12, *ψ* = 0) or its orthogonal counterpart $|\beta_{2}^{+}$> = (*χ* = π/12, *ψ* = π/2). To visualize the polarization states, the selected EP states are represented as certain points on the surface of the Poincaré sphere, as illustrated in Figure 3c. For each wavelength, the different combinations of the incident and output polarizations yield four distinct configurations, corresponding to the four components of the Jones matrix. As demonstrated in Figure 3d,e, under specific wavelength and polarization conditions, both numerical and experimental results successfully achieved the reconstruction of target images across all eight channels. The absolute diffraction efficiency at these two wavelengths are evaluated for both simulated and experimentally reconstructed holograms (see details in Note S4, Supporting Information). The maximum efficiency reaches 20.2% in simulations and 14.5% in experiments. Furthermore, a comparative analysis of efficiency has been conducted between the proposed metasurface and state‐of‐the‐art holographic metasurfaces with single‐, dual‐, and triple‐Jones‐matrix channels (see details in Note S5, Supporting Information). The results demonstrate that our dual‐wavelength and four‐elliptical‐polarization multiplexed meta‐hologram achieves performance comparable to that of state‐of‐the‐art holographic metasurfaces. To perform a quantitative evaluation of the crosstalk, we analyzed the simulated and experimental signal‐to‐noise ratio (SNR) of the reconstructed holographic images (see details in Note S6, Supporting Information). All simulated SNR values exceed 0.39, whereas the experimental SNR values are lower than the simulated ones, with the minimum SNR reaching 0.21. The difference in SNR ratio between the simulation and experimental holograms is primarily attributed to deviations caused by fabrication and errors in measurement. The results validate the successful realization of dispersive full‐channel Jones matrix modulation. To further validate the feasibility of the proposed method, we extended this design strategy to three distinct wavelengths. We demonstrate a meta‐hologram capable of implementing across three wavelengths (specifically, *λ_R_* = 635 nm, *λ_G_* = 532 nm, and a blue (B) wavelength *λ_B_* = 450 nm) and four Jones matrix channels (*J*_11_, *J*_12_, *J*_21_, and *J*_22_), corresponding to a total of twelve independent multiplexing channels. At the working wavelength *λ_B_*, the selected EP bases are $|\alpha_{3}^{+}$> = (*χ* = 5π/12, *ψ* = π/4) and $|\beta_{3}^{+}$> = (*χ* = π/12, *ψ* = 3π/4) (see details in Note S7, Supporting Information). As a result, twelve independent color holographic images can be reconstructed at the imaging plane (the numerical simulation results are provided in Note S8, Supporting Information). While the current design targets discrete wavelengths, the proposed paradigm provides a foundational framework that could be extended to finite bandwidths through advanced optimization techniques and more complex meta‐atom designs. Therefore, the inherent limitations of single‐layer metasurface in multiplexing channel capacity can be effectively overcome through synergistically utilizing synergistic wavelength dispersion engineering and polarization base transformation, thereby establishing a novel paradigm for the development of high‐capacity optical devices.

## 4 Implementation of Dual‐Key‐Space Convolutional Encryption

Metasurfaces provide a brand‐new solution for optical encryption, enabling high‐capacity and high‐security information protection by leveraging multiple DoFs of light. However, conventional encryption strategies based on a single or simple combination of DoFs of light remain vulnerable to brute‐force attacks. To address this issue, our scheme utilizes the full‐channel dispersion‐based Jones matrix modulation capability of a single‐layer metasurface within a dual‐key space convolution framework. Building upon the demonstrated multi‐channel multiplexing capability of the single‐layered metasurface for dispersive full‐channel Jones matrix modulation, we developed an innovative optical encryption scheme with unprecedented security. In this framework, the incident wavelength, incident polarization state, outgoing polarization state, and imaging plane collectively form two distinct sets of encryption keys. These keys govern the generation of target information, thus achieving a significant superiority over conventional single‐DoF encryption methods.

**Figure 4:**
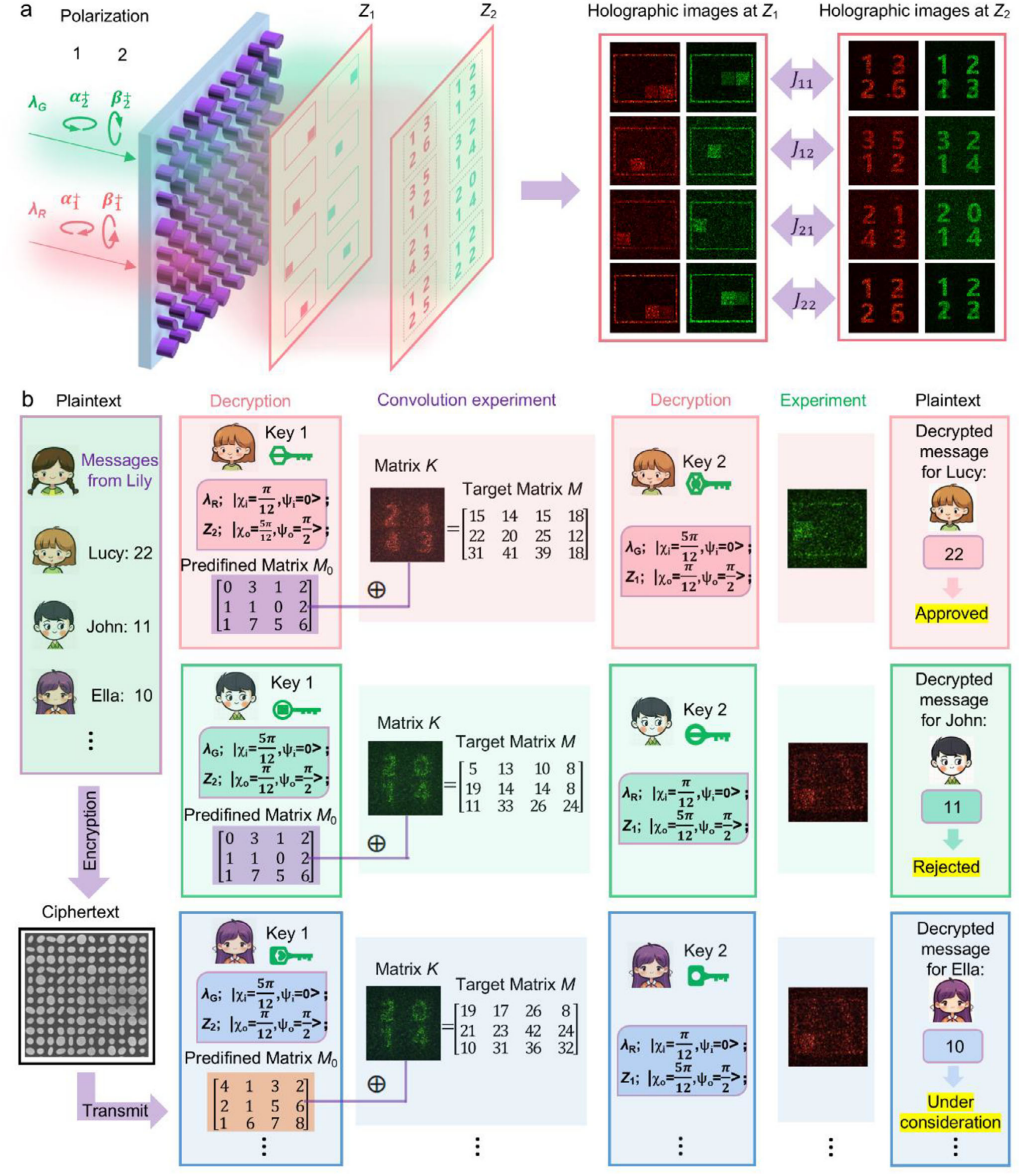
Schematic illustration of the dual‐key‐space convolution encryption platform. a) The schematic configuration of the meta‐device for encoding two sets of holograms used for encryption. b) The conceptual framework of the dual‐key‐space convolution encryption platform. Lily encrypts her messages for Lucy (22), John (11), Ted (10), and so forth, all on a single metasurface. Identical samples are distributed to the respective receivers, who can decrypt their personalized messages using the corresponding customized keys. Each of them holds two sets of keys, both containing customer‐specific parameters, including incident wavelength, incident polarization state, outgoing polarization state, and imaging plane distance. Through convolution operation and extracting the positions of matrix elements, and then combining with the predefined encoding table set by the sender, different encrypted information can be achieved.

**Figure** **4**a illustrates the schematic configuration of the meta‐device employed for dual‐key‐space convolution encryption. Here, we extend the above‐mentioned color vectorial meta‐hologram to 3D space, with sixteen holographic images exhibited at two imaging planes. The meta‐device has been elaborately designed to encode two different types of images within each independent multiplexing channel. When illuminated with light own a specific incident wavelength and polarization state, the output images are then selectively extracted via a polarization state analyzer, enabling the successful reconstruction of two pre‐encoded holographic images at the designated output planes *Z*_1_ and *Z*_2_. The numerical results of the two sets of reconstructed holographic images are presented in Figure 4a, while the corresponding experimental results are provided in the Supporting Information (see details in Note S9, Supporting Information).

Figure 4b demonstrates the conceptual framework of the dual‐key‐space convolution encryption platform. The encryption process involves two key steps: First, the plaintext information is encoded as element values at specific position within target matrix *M*. The *M* is generated by convolving a predefined reference matrix *M*_0_ with a convolution kernel matrix *K*, where the information defining *K* is encoded into the single‐layer metasurface through wavelength and polarization state modulation. Second, using another set of independent wavelengths and polarization states, the spatial position information of each plaintext element in matrix *M* is encrypted, and subsequently encoded onto the same metasurface to serve as the final ciphertext.

The decryption process requires simultaneous access to the metasurface ciphertext, the correct encryption keys, the predefined matrix *M*_0_, the matrix calculation rules, and the encoding table. Both the *M*_0_ and the encoding table can be independently designed by the sender. For instance, in our scheme, the sender defined *M*_0_ as a 4 × 3 matrix based on the content of the ciphertext, while the encoding table contains multiple semantic mappings corresponding to different numerical values, such as “22” representing “Approved”, “11” representing “Rejected”, and “10” representing “Under consideration”. Initially, the first key set (i.e., a designated wavelength and polarization state) is applied to illuminate the metasurface. Subsequently, the transmitted light corresponding to the desired Jones matrix channel is selected to reconstruct matrix *K* on plane *Z*_2_. According to the predefined operation rules, the reconstructed *K* serves as the convolution kernel and is convolved with *M*_0_ to obtain *M*. Thereafter, the second key set is utilized to illuminate the metasurface again and select the corresponding Jones matrix channel, enabling another image reconstruction on plane *Z*_1_. The reconstructed image reveals the spatial position information of the plaintext element within matrix *M*. Based on this reconstructed position map, the corresponding element in the previously obtained *M* is extracted. Finally, referring to the pre‐shared code table, the extracted numerical element value is decoded into meaningful plaintext information.

This scheme facilitates the parallel transmission of multiple encrypted messages to distinct users with high security. As illustrated in Figure 4b, Lily intends to deliver different plaintext messages to multiple recipients, such as Lucy, John, and Ella. These messages are encrypted via the dual‐key‐space convolution framework and encoded into a single‐layer metasurface. The matrix calculation rules, and the encoding table used to map numbers to semantic messages have been previously informed to all users. Identical physical samples, along with matrix *M*_0_ and user‐customized keys, are then distributed to the respective receivers. It is important to note that as long as either the predefined matrix *M*_0_ or the decryption keys differ among users, the final extracted information will be different. For example, Lucy and John hold the same *M*_0_ but different decryption keys. When applying their respective first key sets, they reconstruct different holograms for matrix *K* on plane *Z*_2_ = 600 µm. Convolving these distinct *K* with the same matrix *M*_0_ results in different matrix *M*. Subsequently, when applying their respective second key sets, they reconstruct position maps on plane *Z*_1_ = 400 µm. By extracting elements from their respective *M* based on these maps, Lucy obtains the number “22”, which corresponds to “Approved” in the encoding table; while Tom extracts the number “11”, representing “Rejected”. For Ella, who holds the same decryption keys as John but a different matrix *M*_0_, even if she reconstructs the same holographic images for matrix *K* and position map as John, she extracts the number “10” from her matrix *M*, which corresponds to “Under consideration” in the encoding table.

The proposed dual‐key‐space convolutional encryption platform demonstrates exceptional security performance. This substantial improvement in security stems from its multi‐dimensional decryption mechanism and the strong interdependence among critical components: the metasurface ciphertext, the correct encryption keys, the predefined matrix *M*_0_, the matrix operation rules, and the encoding table. The absence of any single component renders the decryption process infeasible. As a sophisticated physical encryption medium, the metasurface encodes information across multiple dispersive Jones matrix channels and spatial planes, thereby effectively preventing unauthorized extraction or duplication of plaintext data. By incorporating mathematical operations and semantic mapping based on the encoding table, the system introduces additional security layers at both computational and semantic levels. The synergistic integration of physical optical encryption mechanism (including wavelength, polarization state, and spatial DoFs) with computational encryption techniques (such as matrix convolution and element mapping) establishes a formidable security barrier. Furthermore, the scheme theoretically enables infinite channel capacity through wavelength‐dimensional expansion via dispersion‐based full‐channel Jones matrix modulation, in conjunction with flexibly configurable operational rules and predefined matrices. To further validate the efficacy of the proposed method, we presented the simulation results of a three wavelengths full Jones matrix channels holographic display for encryption system, demonstrating twelve distinct convolution kernels *K* in the Z_2_ plane and twelve element position mapping images in the *Z*_1_ plane (see details in Note S10, Supporting Information). These results further confirm that this method can effectively overcome the vulnerabilities existing in traditional single‐parameter encryption schemes, thereby laying a solid foundation for the development of large‐capacity and high‐security optical information processing systems.

## 5 Conclusion

In conclusion, we have demonstrated a general dispersive full‐channel Jones matrix modulation strategy that enables the independent decoupling of all Jones matrix phase channels and the manipulation of wavelength dispersion. The physical mechanism relies on synergistic wavelength dispersion engineering and polarization base transformation. By operating under EP states, the theoretical upper limit on accessible Jones matrix channels in conventional single‐layer metasurface was effectively overcome, enabling simultaneous and independent control of all four Jones matrix channels across multiple discrete wavelengths. As both numerical and experimental validation, a dual‐wavelength vectorial meta‐hologram was implemented, successfully reconstructing eight independent holographic images. To further verify the proposed strategy, we extend the color vectorial meta‐hologram to 3D space, with sixteen holographic images exhibited at two imaging planes. Leveraging the proposed 3D meta‐hologram, we constructed a novel dual‐key‐space convolutional encryption platform. This platform uniquely merges physical optical keys with mathematical operations, significantly enhancing security and parallel data transmission capacity. This work provides a versatile foundational paradigm for developing next‐generation ultra‐compact, high‐capacity optical devices, including advanced multi‐channel displays, ultra‐secure optical encryption systems, and high‐throughput parallel optical information processing platforms.

## 6 Experimental Section

### Metasurface Fabrication

The samples were fabricated using electron beam lithography (EBL) along with the etching technique. First, a 1000‐nm‐thick polymethyl methacrylate (PMMA) electron‐beam resist layer was spin‐coated at 2000 rpm on the transparent silica substrate with an ITO film layer and baked on a hot plate for 4 min at 180 °C. Next, the sample was exposed by EBL with a 100‐KV voltage and a beam current of 200 pA. Subsequently, the exposed sample was put in a mixed solution of isopropanol and methyl isobutyl ketone (IPA: MIBK = 3:1) for 1 min, and then fixed it in the IPA solution for 1 min at room temperature. Later, the atomic layer deposition system was used to fill the exposed area with 200 nm TiO_2_. PMMA was sued for the positive photoresist exposure process, which is void before deposition. Then the deposited thickness of TiO_2_ is related to the semi‐minor axis of the maximum meta‐atom. After this process, there will be a layer of 200 nm TiO_2_ on the top of the entire sample, which was removed by ion beam etching in the next process. After removing the TiO_2_ on the top layer, reactive ion etching was used to remove the resist. Finally, the TiO_2_ nanostructures with a high aspect ratio (of up to 10) are obtained.

## Conflict of Interest

The authors declare no conflict of interest.

## Supporting information



Supporting Information

## Data Availability

The data that support the findings of this study are available from the corresponding author upon reasonable request.
