# Supplementary material for: Dispersive Full‐Channel Jones Matrix Modulation in Elliptical Polarization Bases via a Single‐Layered Metasurface
Source: Adv Sci (Weinh). 2025 Dec 14;13(12):e21354. doi: 10.1002/advs.202521354 (PMC12948192; doi:10.1002/advs.202521354)
Supplement: Supplementary file 1 — Supporting Information [file ADVS-13-e21354-s001.docx]

**Supporting Information**

**Dispersive Full-channel Jones Matrix Modulation in Elliptical Polarization Bases via a Single-layered Metasurface**

*Hairong He^†^, Guangtao Cao^†^, Shuhao Zhang, Hui Xu*, Zhiquan Chen, Meiyu Peng, Yuting Jiang, Yueqiang Hu*, Hui Jing, Huigao Duan, and Hui Yang**

H. He, S. Zhang, M. Peng, H. Jing, and H. Yang
Key Laboratory of Low-Dimensional Quantum Structures and Quantum Control of Ministry of Education, Department of Physics, Hunan Normal University, Changsha 410081, China.
*E-mail：yangh1023@126.com

H. He, and S. Zhang
Key Laboratory of Physics and Devices in Post-Moore Era, College of Hunan Province, Changsha, 410081, China

G. Cao
School of Physics and Electronic Sciences, Changsha University of Science and Technology, Changsha 410004, China

1. Xu, and Z. Chen

School of Microelectronics and Physics, Hunan University of Technology and Business, Changsha 410205, P.R. China

*E-mail：xuhui@hutb.edu.cn

Y. Jiang, Y. Hu, and H. Duan

National Research Center for High-Efficiency Grinding, College of Mechanical and Vehicle Engineering, Hunan University, Changsha 410082, China

*E-mail：huyq@hnu.edu.cn

† These authors contributed equally to this work.

**Supplementary Note 1: Wavelength modulated full Jones matrix channel multiplexing**

Here, for wavelength modulated full-polarization channel multiplexing design, we use a super-unit consists of four meta-atoms, each of which with elliptical cross-sections that exhibit birefringence. The wavelength-modulated Jones matrix of a single meta-atom can be expressed as

$J\left( \lambda\right)=R\left( -\theta\right)[\begin{matrix} e^{i\delta_{x}\left( \lambda\right)} & 0 \\ 0 & e^{i\delta_{y}\left( \lambda\right)} \end{matrix}]R\left( \theta\right)$ (S1)

where *λ* is the working wavelength and $R\left( \theta\right)=[\begin{matrix} cos\theta& sin\theta\\ -sin\theta& cos\theta\end{matrix}]$ is the rotation matrix. $\delta_{x}()$ and $\delta_{y}()$ represent the propagation phase shifts under two orthogonal linearly polarized (LP) lights at wavelength *λ* along the meta-atoms’ two symmetry axes. Here the meta-atoms are treated as the local metasurface and the coupling between the meta-atoms is negligible. For the super-cell, the final dispersive Jones matrix can be expressed as

$$J\left( \lambda\right)=\sum_{m=1}^{4} R\left( -\theta_{m} \right)\left[ \begin{matrix} e^{i\delta_{xm}\left( \lambda\right)} & 0 \\ 0 & e^{i\delta_{ym}\left( \lambda\right)} \end{matrix} \right]R\left( \theta_{m} \right)=\sum_{m=1}^{4} \begin{aligned} \left[ \begin{matrix} {cos}^{2}\left( \theta_{m} \right)e^{i\delta_{xm}\left( \lambda\right)}+{sin}^{2}\left( \theta_{m} \right)e^{i\delta_{ym}\left( \lambda\right)} & \sin\left( \theta_{m} \right)\cos\left( \theta_{m} \right)e^{i\delta_{xm}\left( \lambda\right)}-\sin\left( \theta_{m} \right)\cos\left( \theta_{m} \right)e^{i\delta_{ym}\left( \lambda\right)} \\ \sin\left( \theta_{m} \right)\cos\left( \theta_{m} \right)e^{i\delta_{xm}\left( \lambda\right)}-\sin\left( \theta_{m} \right)\cos\left( \theta_{m} \right)e^{i\delta_{ym}\left( \lambda\right)} & {cos}^{2}\left( \theta_{m} \right)e^{i\delta_{ym}\left( \lambda\right)}+{sin}^{2}\left( \theta_{m} \right)e^{i\delta_{xm}\left( \lambda\right)} \end{matrix} \right] \\ \end{aligned}$$

$=\left[ \begin{matrix} e^{i\delta_{11}\left( \lambda\right)} & e^{i\delta_{12}\left( \lambda\right)} \\ e^{i\delta_{21}\left( \lambda\right)} & e^{i\delta_{22}\left( \lambda\right)} \end{matrix} \right]=\left[ \begin{matrix} J_{11}(\lambda) & J_{12}(\lambda) \\ J_{21}(\lambda) & J_{22}(\lambda) \end{matrix} \right]$ （S2）

where $\delta_{mn}$ (*m*, *n* = 1, 2) are the phase shifts in the four channels of the final superimposed dispersive Jones matrix. Here the amplitudes of the four channels is regarded as unity for simplicity. The Jones matrix in the orthogonal circular polarization bases can be derived as

$$S\left( \lambda\right)=\left[ \begin{matrix} S_{11}(\lambda) & S_{12}(\lambda) \\ S_{21}(\lambda) & S_{22}(\lambda) \end{matrix} \right]$$

=$\left[ \begin{matrix} 1/2[\left( J_{11}\left( \lambda\right)+J_{22}\left( \lambda\right) \right)-i(J_{12}\left( \lambda\right)-J_{21}\left( \lambda\right))] & 1/2[\left( J_{11}\left( \lambda\right)-J_{22}\left( \lambda\right) \right)+i(J_{12}\left( \lambda\right)+J_{21}\left( \lambda\right))] \\ 1/2[\left( J_{11}\left( \lambda\right)-J_{22}\left( \lambda\right) \right)-i(J_{12}\left( \lambda\right)+J_{21}\left( \lambda\right))] & 1/2[\left( J_{11}\left( \lambda\right)+J_{22}\left( \lambda\right) \right)+i(J_{12}\left( \lambda\right)-J_{21}\left( \lambda\right))] \end{matrix} \right]$(S3)

Next, we consider the optical response of the super-cell with an arbitrary elliptical polarized incident light. For an arbitrary pair of orthogonal elliptical polarization states, $|\alpha^{+}>$ and $|\beta^{+}>$, which can be expressed in the orthogonal circular polarization bases as the form

$|\alpha^{+}> =[\begin{matrix} e^{i\psi}cos \\ e^{-i\psi}sin \end{matrix}]$ $|\beta^{+}> =[\begin{matrix} -e^{i\psi}sin \\ e^{-i\psi}cos \end{matrix}]$ (S4)

where $\psi$ and $\chi$ represent the azimuth angle and ellipticity of the polarization ellipse, respectively. For this scenario, we can detect the co-polarization or cross-polarization by placing both a polarizer and quarter wave-plate pairs before and after the metasurface. The optical response, i.e., the modulation of dispersive full Jones matrix polarization channels, can be written as

$P_{11}\left( \lambda\right)=\left( {|\alpha^{+}>}^{*} \right)^{T}S(\lambda)|\alpha^{+}>$

$={cos}^{2}()S_{11}\left( \lambda\right)+e^{-i2\psi}\frac{sin(2)}{2}S_{12}\left( \lambda\right)+e^{i2\psi}\frac{sin(2)}{2}S_{21}\left( \lambda\right)+{sin}^{2}()S_{22}\left( \lambda\right)$ (S5)

$P_{12}\left( \lambda\right)=\left( {|\alpha^{+}>}^{*} \right)^{T}S(\lambda)|\beta^{+}>$

$=-\frac{sin(2)}{2}S_{11}\left( \lambda\right)+{cos}^{2}()S_{12}\left( \lambda\right){-e}^{i2\psi}{sin}^{2}()S_{21}\left( \lambda\right)+e^{i2\psi}\frac{sin(2)}{2}S_{22}\left( \lambda\right)$ (S6)

$P_{21}\left( \lambda\right)=\left( {|\beta^{+}>}^{*} \right)^{T}S(\lambda)|\alpha^{+}>$

$=-\frac{sin(2)}{2}S_{11}\left( \lambda\right)-e^{-i2\psi}{sin}^{2}({)S}_{12}\left( \lambda\right){+e}^{i2\psi}{cos}^{2}()S_{21}\left( \lambda\right)-\frac{sin(2)}{2}S_{22}\left( \lambda\right)$ (S7)

$P_{22}\left( \lambda\right)=\left( {|\beta^{+}>}^{*} \right)^{T}S(\lambda)|\beta^{+}>$

$={sin}^{2}()S_{11}\left( \lambda\right){-e}^{i2\psi}\frac{sin(2)}{2}S_{12}\left( \lambda\right)-e^{i2\psi}\frac{sin(2)}{2}S_{21}\left( \lambda\right)+{cos}^{2}(\psi)S_{22}\left( \lambda\right)$ (S8)

Mathematically, Eqs. (S5-S8) can be expressed as the matrix form as

$\left[ \begin{matrix} P_{11}(\lambda) \\ P_{12}(\lambda) \\ \begin{matrix} P_{21}(\lambda) \\ P_{22}(\lambda) \end{matrix} \end{matrix} \right]=\left[ \begin{matrix} \begin{matrix} {cos}^{2}() & e^{-i2\psi}\frac{sin(2)}{2} \\ -\frac{sin(2)}{2} & {cos}^{2}() \end{matrix} & \begin{matrix} e^{i2\psi}\frac{sin(2)}{2} & {sin}^{2}() \\ {-e}^{i2\psi}{sin}^{2}() & e^{i2\psi}\frac{sin(2)}{2} \end{matrix} \\ \begin{matrix} -\frac{sin(2)}{2} & -e^{-i2\psi}{sin}^{2}() \\ {sin}^{2}() & {-e}^{-i2\psi}\frac{sin(2)}{2} \end{matrix} & \begin{matrix} e^{i2\psi}{cos}^{2}() & -\frac{sin(2)}{2} \\ -e^{i2\psi}\frac{sin(2)}{2} & {cos}^{2}() \end{matrix} \end{matrix} \right]\left[ \begin{matrix} S_{11}\left( \lambda\right) \\ S_{12}\left( \lambda\right) \\ \begin{matrix} S_{21}\left( \lambda\right) \\ S_{22}\left( \lambda\right) \end{matrix} \end{matrix} \right]$ (S9)

This equation can be simplified as $P\left( \lambda\right)=M(\lambda)\cdot S(\lambda)$, where $P\left( \lambda\right)={[P_{11}\left( \lambda\right),P_{12}\left( \lambda\right),P_{21}\left( \lambda\right)，P_{22}(\lambda)]}^{T}$, $S\left( \lambda\right)={[S_{11}\left( \lambda\right),S_{12}\left( \lambda\right),S_{22}\left( \lambda\right),S_{22}\left( \lambda\right)]}^{T}$, and $M(\lambda)$ is a 4×4 matrix. For a meta-device operates simultaneously at *N* discrete wavelengths, Eq. (S9) can be expressed as

$$\left[ \begin{matrix} P_{11}(\lambda_{1}) \\ P_{12}(\lambda_{1}) \\ \begin{matrix} P_{21}(\lambda_{1}) \\ P_{22}(\lambda_{1}) \end{matrix} \end{matrix} \right]\ldots\left[ \begin{matrix} P_{11}(\lambda_{N}) \\ P_{12}(\lambda_{N}) \\ \begin{matrix} P_{21}(\lambda_{N}) \\ P_{22}(\lambda_{N}) \end{matrix} \end{matrix} \right]=$$

$\left[ \begin{matrix} \begin{matrix} {cos}^{2}() & e^{-i2\psi}\frac{sin(2)}{2} \\ -\frac{sin(2)}{2} & {cos}^{2}() \end{matrix} & \begin{matrix} e^{i2\psi}\frac{sin(2)}{2} & {sin}^{2}() \\ {-e}^{i2\psi}{sin}^{2}() & e^{i2\psi}\frac{sin(2)}{2} \end{matrix} \\ \begin{matrix} -\frac{sin(2)}{2} & -e^{-i2\psi}{sin}^{2}() \\ {sin}^{2}() & {-e}^{-i2\psi}\frac{sin(2)}{2} \end{matrix} & \begin{matrix} e^{i2\psi}{cos}^{2}() & -\frac{sin(2)}{2} \\ -e^{i2\psi}\frac{sin(2)}{2} & {cos}^{2}() \end{matrix} \end{matrix} \right]\left[ \begin{matrix} S_{11}(\lambda_{1}) \\ S_{12}(\lambda_{1}) \\ \begin{matrix} S_{21}(\lambda_{1}) \\ S_{22}(\lambda_{1}) \end{matrix} \end{matrix} \right]\ldots\left[ \begin{matrix} S_{11}\left( \lambda_{N} \right) \\ S_{12}\left( \lambda_{N} \right) \\ \begin{matrix} S_{21}\left( \lambda_{N} \right) \\ S_{22}\left( \lambda_{N} \right) \end{matrix} \end{matrix} \right]$ (S10)

**Supplementary Note 2: The simulation database on propagation phases and transmittance of the meta-atom**

Here, we displayed the simulation database on propagation phases and transmittance of the meta-atom as a function of nanoblock’s in-plane dimensions (*L_x_* and *L_y_*) under *x*-linearly and *y*-linearly polarized incident light with wavelengths of *λ*_R_, *λ*_G_ and *λ*_B_, as shown in Fig. S1 and S2, respectively. We employed the finite-difference time-domain (FDTD) method for full-wave simulation. Periodic boundary conditions were applied in the *x* and *y* directions to model an infinitely extended periodic structure, whereas a perfectly matched layer (PML) was implemented in the *z* direction to effectively absorb electromagnetic waves and replicate the propagation conditions of an open space. The meta-atoms must be meticulously designed to precisely control the propagation phase, ensuring complete coverage of the 0 to 2π range. Based on the constructed propagation phase database, super cells consisting of four meta-atoms involving 12 independent variables (i.e., [*L_xk_*, *L_yk_*, *θ_k_*], k = 1, 2, 3, 4) are used to solve each objective functions. In this case, the relation between Jones matrix elements and variables of the super cell was established, thereby achieving the holographic function of multi-channel multiplexing.


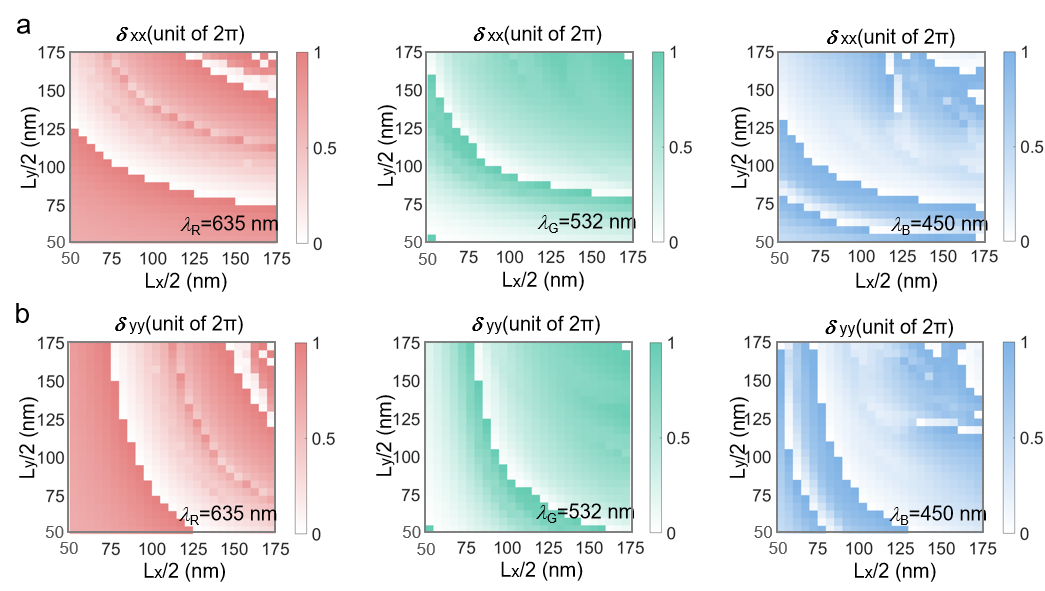


**Figure S1.** The simulation database on propagation phases of the meta-atom as a function of nanoblock’s in-plane dimensions (*L_x_* and *L_y_*) under (a) *x*-linearly and (b) *y*-linearly polarized incident light with wavelengths of *λ_R_* = 635 nm, *λ_G_* = 532 nm and *λ_B_* = 450 nm.


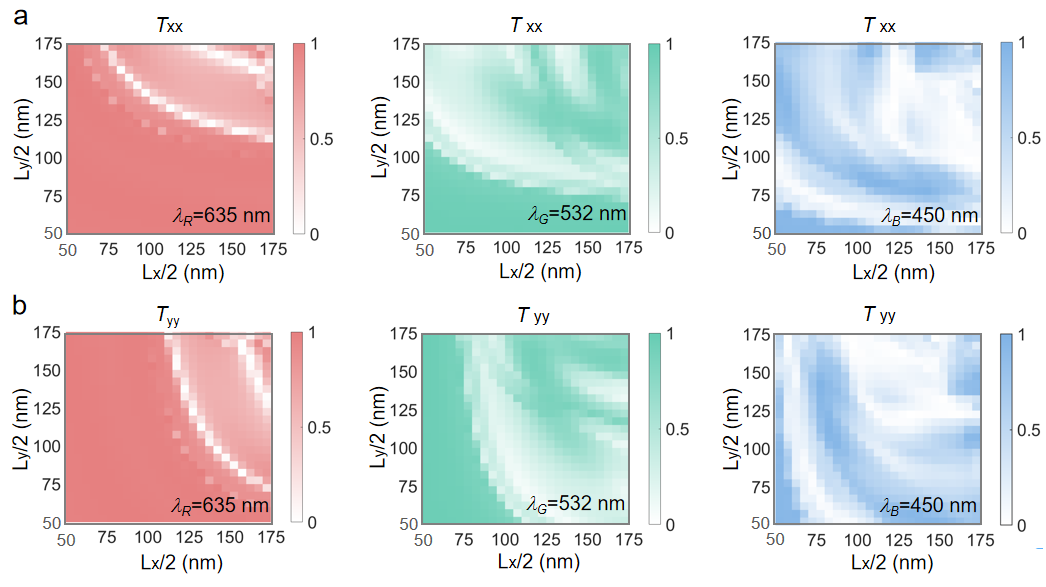


**Figure S2.** The simulation database on transmittance of the meta-atom as a function of nanoblock’s in-plane dimensions (*L_x_* and *L_y_*) under (a) *x*-linearly and (b) *y*-linearly polarized incident light with wavelengths of *λ_R_* = 635 nm, *λ_G_* = 532 nm and *λ_B_* = 450 nm.

**Supplementary Note 3:** **Experimental setup for characterizing the meta-device**

Figure S3 illustrates the schematic diagram of the experimental setup used for the optical measurement of the fabricated metasurface devices intended for meta-holograms. The required monochromatic light is obtained by passing the output of a continuous-wave laser source through a spectral filter. The desired elliptical polarization states of the input light are generated by a polarization state generator (PSG), which is composed of a linear polarizer (LP) and a quarter-wave plate (QWP). Subsequently, the polarized light is normally incident on the metasurface. The light scattered by the metasurface was collected by a objective and further isolated using a polarization state analyzer (PSA), which comprises another QWP and LP. Finally, the the intensity pattern is captured by a charge-coupled device (CCD) camera.


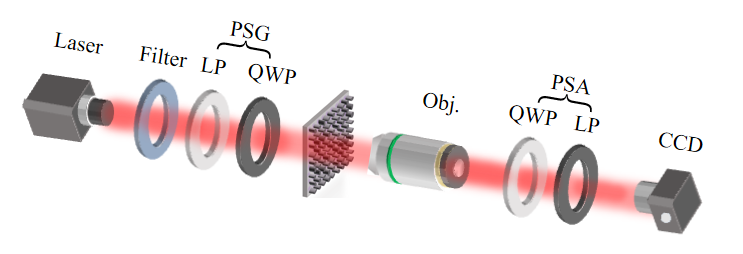


**Figure S3.** Optical setup for the measurement of meta-holograms. The desired elliptical polarization states of the input light are generated by a polarization state generator (PSG), which is composed of a linear polarizer (LP) and a quarter-wave plate (QWP). Subsequently, the polarized light is normally incident on the metasurface.

**Supplementary Note 4: Absolute diffraction efficiency of the metasurface**

Table S1 Absolute diffraction efficiency of the metasurface at RG wavelengths

| Working wavelengths | *λ*_G_ =532 nm | | | | *λ*_R_ =635 nm | | | |
| --- | --- | --- | --- | --- | --- | --- | --- | --- |
| Jones matrix channels | *J*_11_ | *J*_12_ | *J*_21_ | *J*_22_ | *J*_11_ | *J*_12_ | *J*_21_ | *J*_22_ |
| Simulated efficiency | 19.8 % | 18.4 % | 18.2 % | 19.7 % | 20.1 % | 18.8 % | 19.0 % | 20.2 % |
| Experimental efficiency | 14.0 % | 14.3 % | 14.1% | 13.1 % | 13.9 % | 14.4 % | 14.5% | 14.2 % |

Table S1 shows the absolute diffraction efficiency of the metasurface at the two operating wavelengths *λ*_R_ = 635 nm and *λ*_G_ = 532 nm. Here, efficiency is defined as the ratio of the measured power of holographic images to the total optical power incident on the effective area of the component. The maximal simulated efficiency reached 20.2%. In actual experiments, due to the unavoidable fabrication and the measurement errors, the experimental efficiencies are lower than the simulated values, with a maximum of 14.5%.

**Supplementary Note 5: Efficiency comparisons of state-of-the-art holographic metasurfaces**

Table S2 Efficiency comparison of state-of-the-art holographic metasurfaces

| Reference | Working wavelength | Jones matrix channels | Maximal calculated efficiency | Maximal measured efficiency |
| --- | --- | --- | --- | --- |
| [1] | 825 nm | 1 | 94% | 80% |
| [2] | 780 nm | 2 | - | 18% |
| [3] | 671 nm. | 3 | - | 15.1% |
| [4] | 532 nm, 650nm, 715nm, 808nm,  860 nm. | 3 | 33% | 25% |
| This work | 532 nm,  635 nm | 4 | 20.2% | 14.5% |

**Supplementary Note 6: SNR for the holographic images of the metasurface**

Table S3 The simulated and experimental SNR for the holographic images

| Working wavelengths | *λ*_G_=532 nm | | | | | *λ*_R_*=*635 nm | | | |
| --- | --- | --- | --- | --- | --- | --- | --- | --- | --- |
| Jones matrix channels | *J*_11_ | *J*_12_ | *J*_21_ | *J*_22_ | *J*_11_ | | *J*_12_ | *J*_21_ | *J*_22_ |
| Simulated SNR | 0.39 | 0.69 | 0.66 | 0.43 | 0.45 | | 0.60 | 0.62 | 0.49 |
| Experimental SNR | 0.36 | 0.51 | 0.50 | 0.36 | 0.30 | | 0.42 | 0.39 | 0.21 |

Table S3 presents the SNR for both simulated and experimentally measured holographic imageswe performed a quantitative evaluation of the crosstalk between the different polarization and wavelength channels. The signal-to-noise ratio (SNR) are defined as $SNR=\frac{\frac{1}{M\times N}\sum_{i=1}^{M} \sum_{j=1}^{N} O_{ij}}{\sqrt{\frac{!}{M\times N}\sum_{i=1}^{M} \sum_{j=1}^{N} ({O_{ij}-I_{ij})}^{2}}}$, where *I* and *O* represent the intensity of the reconstructed (output) image and the target image, respectively. M and *N* are the size of the image.^[4]^

**Supplementary Note 7: The spatial distributions of the three sets of elliptically polarization bases**


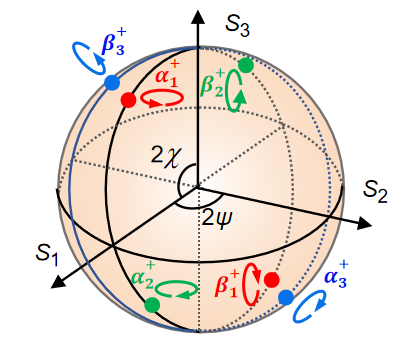


**Figure S4.** The spatial distributions of the three sets of elliptically polarization bases on the Poincaré sphere at the three designated wavelengths (*λ_R_* = 635 nm, *λ_G_* = 532 nm and *λ_B_* = 450 nm) enable the effective decoupling of the four phase channels within the full Jones matrix.

**Supplementary Note 8: Numerical results of the dispersive full-channel Jones matrix meta-hologram at three wavelengths**


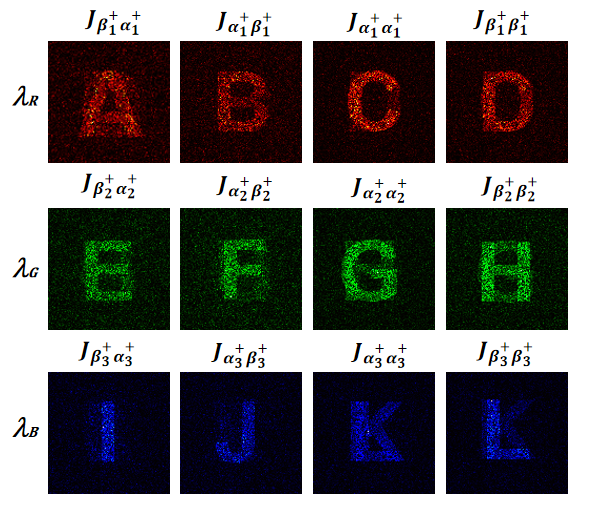


**Figure S5.** The numerical simulation results demonstrate the holographic display performance across three wavelengths (*λ_R_* = 635 nm, *λ_G_* = 532 nm and *λ_B_* = 450 nm) and four Jones matrix channels, corresponding to a total of twelve independent multiplexing channels.

**Supplementary Note 9: Experimentally results of the two wavelengths three-dimensional meta-hologram**


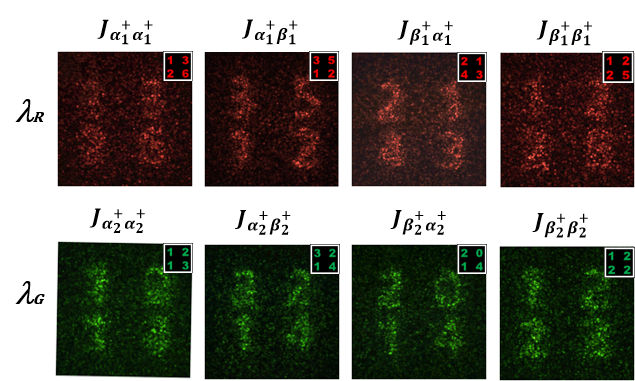


**Figure S6.** The experimentally obtained reconstruction results of the eight independent vectorial holograms associated with the convolution kernel matrix *K* for the two-wavelength four-Jones matrix channels dual-key space convolution encryption platform measured on plane *Z*_2_=600 μm. The inserts show the eight target images, respectively.


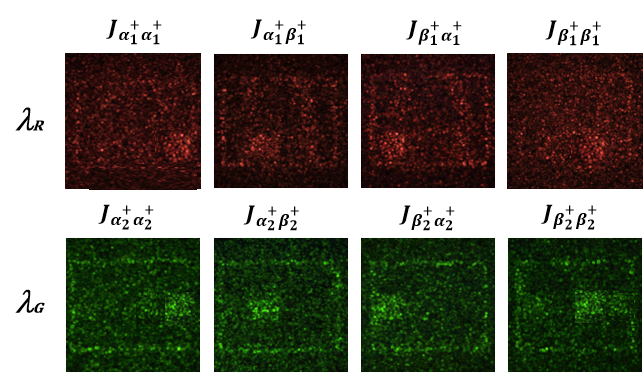


**Figure S7.** The experimentally obtained reconstruction results of the eight independent vectorial holograms associated with the element position map for two-wavelength four-Jones matrix channels dual-key space convolution encryption platform, which were measured on plane *Z*_1_=400 μm.

**Supplementary Note 10: Numerical results of the three wavelengths full Jones matrix channels meta-hologram**


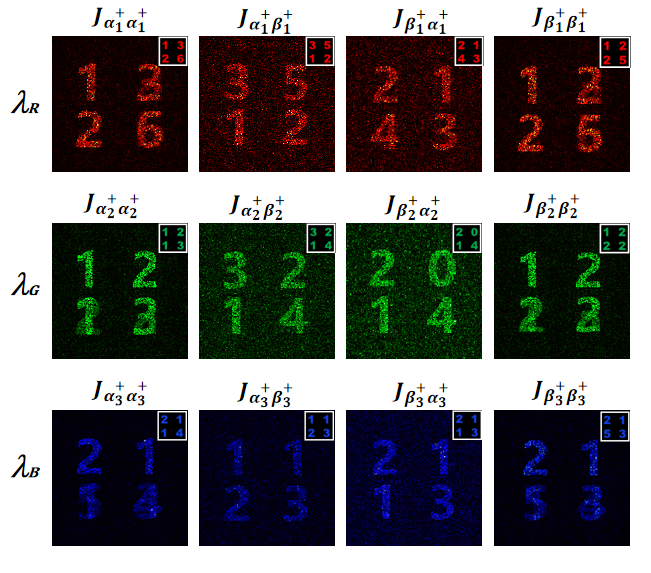


**Figure S8.** The numerical simulation reconstruction results of the twelve independent vectorial holograms associated with the convolution kernel matrix *K* for the three-wavelength four-Jones matrix channels dual-key space convolution encryption platform measured on plane *Z*_2_=600 μm. The inserts show the eight target images, respectively.


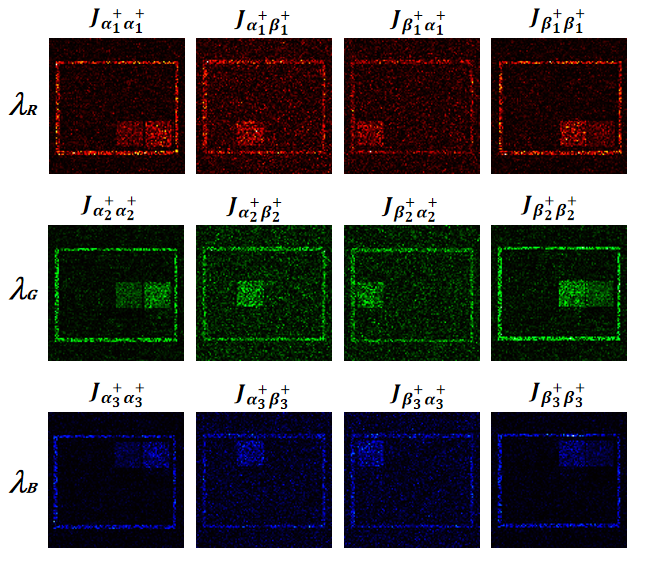


**Figure S9.** The numerical simulation reconstruction results of the twelve independent vectorial holograms associated with the element position map for three-wavelength four-Jones matrix channels dual-key space convolution encryption platform, which were measured on plane *Z*_1_=400 μm.

**References**

1. G. Zheng, H. Mühlenbernd, M. Kenney, G. Li, T. Zentgraf, and S. Zhang, “Metasurface holograms reaching 80% efficiency,” Nature Nanotechnology **10**(4), 308-312 (2015).
2. W. Chen, K. Yang, C. Wang, Y. Huang, G. Sun, I. Chiang, C. Liao, W. Hsu, H. Lin, S. Sun, L. Zhou, A. Liu, and D. Tsai, “High-efficiency broadband meta-hologram with polarization-controlled dual images,” Nano Letters **14**(1), 225-230 (2014).
3. Y. Bao, L. Wen, Q. Chen, C.-W. Qiu, and B. Li, “Toward the capacity limit of 2D planar Jones matrix with a single-layer metasurface,” Science Advances **7**, eabh0365 (2021).
4. Y. Bao, H. Shi, R. Wei, B. Wang, Z. Zhou, Y. Chen, C.-W. Qiu, and B. Li, “Efficient Gradient-Based Metasurface Optimization toward the Limits of Wavelength-Polarization Multiplexing,” Nano Letters **25**, 6340-6347 (2025).
